# Mesenchymal stem cell pretreatment of non-heart-beating-donors in experimental lung transplantation

**DOI:** 10.1186/s13019-014-0151-3

**Published:** 2014-09-02

**Authors:** Thorsten Wittwer, Parwis Rahmanian, Yeong-Hoon Choi, Mohamed Zeriouh, Samira Karavidic, Klaus Neef, Astrid Christmann, Tanja Piatkowski, Anke Schnapper, Matthias Ochs, Christian Mühlfeld, Thorsten Wahlers

**Affiliations:** Department of Cardiothoracic Surgery, Heart Center, University of Cologne, Kerpener Strasse 61, Cologne, 50924 Germany; Center of Molecular Medicine, University of Cologne, Cologne, Germany; Institute of Functional and Applied Anatomy, Hannover Medical School, Biomedical Research in Endstage and Obstructive Lung Disease Hannover (BREATH), Member of the German Center for Lung Research (DZL), Hannover, Germany; Cluster of Excellence Rebirth (From Regenerative Biology to Reconstructive Therapy), Hannover, Germany

**Keywords:** Non-heart-beating donors, Perfadex lung preservation, Mesenchymal stem cell therapy, Ischemia-reperfusion injury, Donor pretreatment

## Abstract

**Background:**

Lung transplantation (LTx) is still limited by organ shortage. To expand the donor pool, lung retrieval from non-heart-beating donors (NHBD) was introduced into clinical practice recently. However, primary graft dysfunction with inactivation of endogenous surfactant due to ischemia/reperfusion-injury is a major cause of early mortality. Furthermore, donor-derived human mesenchymal stem cell (hMSC) expansion and fibrotic differentiation in the allograft results in bronchiolitis obliterans syndrome (BOS), a leading cause of post-LTx long-term mortality. Therefore, pretreatment of NHBD with recipient-specific bone-marrow-(BM)-derived hMSC might have the potential to both improve the postischemic allograft function and influence the long-term development of BOS by the numerous paracrine, immunomodulating and tissue-remodeling properties especially on type-II-pneumocytes of hMSC.

**Methods:**

Asystolic pigs (n = 5/group) were ventilated for 3 h of warm ischemia (groups 2–4). 50x106 mesenchymal-stem-cells (MSC) were administered in the pulmonary artery (group 3) or nebulized endobronchially (group 4) before lung preservation. Following left-lung-transplantation, grafts were reperfused, pulmonary-vascular-resistance (PVR), oxygenation and dynamic-lung-compliance (DLC) were monitored and compared to control-lungs (group 2) and sham-controls (group 1). To prove and localize hMSC in the lung, cryosections were counter-stained. Intra-alveolar edema was determined stereologically. Statistics comprised ANOVA with repeated measurements.

**Results:**

Oxygenation (p = 0.001) and PVR (p = 0.009) following endovascular application of hMSC were significantly inferior compared to Sham controls, whereas DLC was significantly higher in endobronchially pretreated lungs (p = 0.045) with overall sham-comparable outcome regarding oxygenation and PVR. Stereology revealed low intrapulmonary edema in all groups (p > 0.05). In cryosections of both unreperfused and reperfused grafts, hMSC were localized in vessels of alveolar septa (endovascular application) and alveolar lumen (endobronchial application), respectively.

**Conclusions:**

Preischemic deposition of hMSC in donor lungs is feasible and effective, and endobronchial application is associated with significantly better DLC as compared to sham controls. In contrast, transvascular hMSC delivery results in inferior oxygenation and PVR. In the long term perspective, due to immunomodulatory, paracrine and tissue-remodeling effects on epithelial and endothelial restitution, an endobronchial NHBD allograft-pretreatment with autologous mesenchymal-stem-cells to attenuate limiting bronchiolitis-obliterans-syndrome in the long-term perspective might be promising in clinical lung transplantation. Subsequent work with chronic experiments is initiated to further elucidate this important field.

**Electronic supplementary material:**

The online version of this article (doi:10.1186/s13019-014-0151-3) contains supplementary material, which is available to authorized users.

## Background

Although lung transplantation has been proven to be an effective standard therapy for patients with different end-stage pulmonary diseases, significant scarcity of suitable donor organs [1] still limits an unrestricted application. Lung retrieval from non-heart-beating donors (NHBD) offers the potential to increase the number of available organs significantly and was shown to result in excellent experimental results [2],[3]. Meanwhile, this approach is established in clinical practice in different countries [3]-[6]. However, primary graft dysfunction due to ischemia/reperfusion-(I/R)-injury is still a major cause of early mortality and morbidity following pulmonary transplantation [7], and bronchiolitis obliterans syndrome (BOS) is a leading cause of long-term mortality in lung transplant recipients [8]. Therefore, donor pretreatment strategies [9] especially in Extended Criteria Donors [10],[11] or NHBD lungs [12],[13] become continuously important to attenuate the deleterious I/R-injury which is known to be associated with both inactivation of intraalveolar surfactant [14]–[16] and damage to the integrity of surfactant-producing alveolar epithelial type II (AE2-) cells [17]. In the latter context it was shown that the beneficial role of hMSC transplantation into the injured lung may be partly mediated by differentiation of hMSC into AE2-cells [18],[19]. Furthermore, growing evidence exists that donor-derived tissue-specific mesenchymal stem cell (hMSC) expansion and fibrotic differentiation is associated with development of BOS in human lung allografts [20],[21]. Therefore, a cell-based pretreatment strategy of the NHBD with recipient-specific hMSC appears to be very attractive and might have the potential to both improve the postischemic surfactant function and influence the long-term development of BOS by the numerous paracrine, immunomodulating and tissue-remodeling properties of hMSC [18],[22]–[24]. Obviously, the variable degree of homing capacity and homing duration are crucial parameters for the extent of positive hMSC effects [25],[26], therefore successful intrapulmonary deposition to facilitate migration of delivered MSC seem to be important factors. So far, however, no comparative studies exist whether a vascular or endobronchial application of exogenous hMSC into the NHBD lung is superior in order to facilitate the postulated beneficial effects. Therefore it was the aim of this preliminary study to evaluate the impact of both delivery routes on success of intrapulmonary deposition and short-term postischemic outcome following experimental lung transplantation. Generally, intra-allograft deposition of recipient-derived hMSC might represent a novel and promising strategy to further optimise early and long-term function also of the pulmonary allograft by specific cell therapy as it was recently shown in hepatic transplantation [27].

## Methods

### Bone-marrow-derived human mesenchymal stem cells (hMSC)

As the isolation and large scale expansion of porcine MSC is not established, bone marrow was harvested from consecutive patients scheduled for elective coronary artery bypass grafting surgery. The institutional ethics committee of the University Hospital Cologne approved the procedure of bone marrow aspiration for this study and patients gave written informed consent. Immediately before sternotomy up to 40 ml of sternal bone marrow (BM) aspirate was collected using a bone marrow harvest needle (Gallini Inc., Mantova, Italy) and heparinized syringes. Bone marrow derived hMSC were isolated, cultured and confirmed as previously described by our group [28]. Briefly, the aspirates were filtered, mononuclear cells were isolated by density gradient centrifugation and seeded at a density of 10^6^ cells per cm^2^ in T75 cell culture flasks (BD) in specialized hMSC media (PAN Biotech, Aidenbach, Germany) supplemented with penicillin/streptomycin (1:100, Life Technologies, Darmstadt, Germany). Cells were grown at 37°C and 5% CO_2_ in a humidified incubator (Binder, Tuttlingen, Germany) and passaged when confluent. To generate cell numbers sufficient for large animal cell transplantations, 1-5x10^6^ hMSC were seeded into multilayered cell culture vessels (Corning, Wiesbaden, Germany; total cell culture surface area: 3.180 cm^2^) and cultured for 14 days. One day before harvesting, hMSC were labeled with fluorescent, paramagnetic microbeads (Bangs Laboratories, Fishers, IN, USA) by incubation overnight. For harvesting, cells were washed three times with PBS (Life Technologies) and detached with 0.05% trypsin (Life Technologies). After assessing total cell number using a hemacytometer the cells were labeled with the fluorescent vital stain DiI (Life Technologies) according to the manufacturer’s instructions and then resuspended in PBS at a concentration of 5 million cells per ml. The total number of hMSC for transplantation was 5x10^7^ (2.27-2.5 million hMSC per kilogram of body weight) according to current evidence in the literature [27],[29].

All hMSC were phenotypically validated by flow cytometry using a FACS Calibur (BD) as published by our group previously (28). Samples (2x10^5^ cells) were stained with fluorochrome-conjugated (phycoerythrin, PE; fluorescein isothiocyanate, FITC) antibodies CD45-FITC (Miltenyi Biotec, Bergisch Gladbach, Germany), CD73-PE, CD90-FITC (BD), CD105-PE (Beckmann Coulter, Krefeld, Germany) and matching isotype controls at recommended dilutions. At least 100.000 cells were included in the data analysis using MACSquantify software v2.5 (Miltenyi Biotec).

### Large animal single lung transplantation model

Female domestic pigs weighing from 20–22 kg were randomized into 4 groups of 5 donor animals, each with another 5 animals in each group weighing from 28–32 kg as organ recipients. In order to prevent a limiting size-mismatch, donor animals were chosen to be somewhat smaller as compared to the recipients. One group served as a sham-operated control group (group 1) and was prone to surgical dissection of hilar structures, but neither organ preservation nor transplantation were performed. In the 3 NHBD test groups, asystolic pigs were ventilated for 3 h of warm ischemic time (groups 2–4). No approval codes on animal research needed to be obtained according to the corresponding ethical committee.

### Surgical procedure

- *Donor preparation*
  All animals were premedicated with ketamine 10% (20 mg/kg), atropine (0.04 mg/kg) and propofol (3 mg/kg). Pigs were then put in the supine position, intubated and mechanically ventilated with 50% oxygen in a pressure-controlled mode with a peak inspiratory pressure of 20 mmHg, a rate of 18 breaths per minute, an inspiratory/exspiratory ratio of 1:1 and a PEEP of 8 mmHg. Anesthesia was continued with infusion of fentanyl (0,3 μg/kg/min), midazolam (20 μg/kg/min) and pancuronium (10 μg/kg/min). All animals received 200 IU/kg of heparin intravenously. A median sternotomy was performed and the pericardium was opened longitudinally. A perfusion cannula with a sideport to measure the perfusion pressure was placed through the auricle into the left atrium. In the NHBD groups 2, 3 and 4, cardiac fibrillation was induced electrically, and the cadaver was ventilated at an F_i_O_2_ of 0.5 and left at room temperature 3 hours of warm ischemic time. In groups 3 and 4 only, 50x10^6^ human bone-marrow-derived mesenchymal stem cells (hMSC) in 10 ml of normal saline were administered during the final 10-min of NHBD-ventilation using either the pulmonary artery (group 3) or were nebulized endobronchially (group 4) using a mobile ultrasonic nebulizer (Nebu-tec GmbH, Elsenfeld, Germany). This nebulizer was connected to the inspiratory limb of the ventilator system and can be used for all types of currently available clinical ventilators. Antegrade perfusion via the pulmonary artery was started directly after hMSC application, and an 8-minute period was required to infuse 1800 ml LPD solution at 4°C with a maximum flushing pressure of 14 mmHg. Ventilation was continued throughout the entire perfusion period. After completion of the preservation, the heart-lung bloc was excised with both lungs inflated in an endinspiratory state and stored at 4°C for 3 hours.
- *Recipient/Sham preparation*
  The anesthetic regimen was identical to the donor procedure. A Swan-Ganz catheter (Baxter Healthcare Corp., Irvine, CA, USA) and a catheter to monitor the arterial pressure (Vygon, France) were placed into the internal jugular vein and right carotid artery, respectively. All animals were placed in a right decubitus position, and a left thoracotomy was performed in the fifth intercostal space. The pulmonary bifurcation, left main bronchus and left pulmonary veins were dissected in all groups. After clamping of the left pulmonary artery and bronchus, the left pulmonary veins were ligated and pneumonectomy was performed in the NHBD groups 2–4 only. The left donor lung was isolated from the heart-lung bloc and prepared for implantation with a large atrial cuff and full length of both the pulmonary artery and left main bronchus. Implantation of the donor lung started with the bronchial anastomosis using a running suture with 4–0 Prolene (Ethicon Inc., Somerville, NJ, USA) followed by the arterial anastomosis with a running suture of 6–0 Prolene. After clamping of the left atrium, a recipient atrial cuff was designed and anastomosed to the donor atrial cuff using a running suture of 5–0 Prolene. Prior to reperfusion, the donor lung was carefully de-aired. The pulmonary artery was then unclamped and the graft reventilated in a pressure controlled mode with a peak inspiratory pressure of 30 mmHg using a PEEP of 10 mmHg with a respiratory rate of 18/min. After 15 minutes of reperfusion, the previously dissected contralateral right pulmonary artery and bronchus were clamped with a vascular clamp both in the sham-operated control-group and all test groups. All lungs were reperfused for 4 hours followed by termination of the experimaent by intravenous injection of magnesium sulfate.

### Functional analysis

In all experiments, arterial and pulmonary artery pressures as well as central venous and left atrial pressures were recorded continuously. Dynamic lung compliance was monitored by the ventilator (Dräger Medical Inc., Lübeck, Germany). An arterial and mixed-venous blood gas analysis was performed initially and in 30 minute intervals during the reperfusion period. Cardiac output was measured continuously by the Swan-Ganz catheter, and systemic and pulmonary vascular resistances were calculated.

### Wheat-germ-agglutinin (WGA) labelling of cryo sections

Samples of the native right lung – after hMSC application according to the respective group, flush preservation with LPD solution and cold storage of 3 hours – were directly fixed by vascular perfusion of 4% paraformaldehyde, were washed in 1 M PBS-Dulbecco (Biochrom AG; L182-50) for 3 days at 4°C and were incubated in O.C.T. compound (Sakura Tissue-Tek; 4583) in cryo molds (Sakura Tissue-Tek; 4566) for 4 hours at room temperature. Thereafter, the samples were frozen in 2-methylbutane (Roth; 3927.1) on dry ice and 6 μm thin cryo sections were made on a Leica CM 3050’S microtome. After drying, the slices were labelled with WGA-FITC (Sigma; L4895) 1:150 in PBS including 5% BSA (Serva; 11930) for 1 hour and the nuclei were stained with DAPI (Molecular Probes; D1306) for additional 15 minutes at room temperature. After washing with PBS images were taken on a Zeiss Axiophot with the F46-00 ET-Set filter for DAPI, the F36-525 HC-Set filter for WGA and the F36-503 HC-Set filter for MSCs from AHF Analysetechnik (Tuebingen, Germany) and processed with Photoshop CS6.

### Fixation, sampling and processing of the left lung allograft

At the end of the reperfusion, biopsy samples (approx. 0.5 cm^3^) were acquired from the upper lobe of the left lung. These samples were immediately embedded in O.C.T. compound (Sakura Tissue-Tek, Staufen, Germany) and snap frozen in 2-methylbutane (Roth, Karlsruhe, Germany) on dry ice. Cryo sections (10 μm) were prepared using a CM1950 cryotome (Leica, Wetzlar, Germany), stained with 4’,6-diamidino-2-phenylindole (DAPI, Life Technologies, Darmstadt, Germany) and microscopically analyzed (Ti-U microscope and NIS 3.4 software, Nikon, Düsseldorf, Germany). Then, the entire left lung was fixed by perfusion with 2 l of a fixative containing 4% formaldehyde (prepared from freshly depolymerized paraformaldehyde) and 0.1% glutaraldehyde in 0.2 M Hepes buffer. Perfusion pressure was 15 cm H_2_O. During fixation, inflation of the lung was maintained at a constant pressure of 12 cm H_2_O. Afterwards, the fixed lung was excised, the volume was estimated by fluid displacement based on buoyancy [29],[30] and the lung was then cut into slices of 2 cm thickness. To ensure that every part of the lung had an equal chance of being included in the analysis, systematic uniform random sampling was performed by projecting a transparent uniform point grid on each tissue slice [31]. Whenever a point hit the surface of a lung slice a tissue block of approximately 1 cm^3^ was excised at the given location and stored in fresh and cold fixative [14]. The samples were subsequently washed in 0.1 M sodium cacodylate, postfixed in osmium tetroxide, washed again in sodium cacodylate and distilled water, stained en bloc in half-saturated watery uranyl acetate over night, dehydrated in an ascending acetone series and finally embedded in a glycol methacrylate resin (Technovit 8100, Heraeus Kulzer, Wehrheim, Germany). Sections of 1.5 μm thickness were cut from the tissue blocks and stained with methylene blue.

### Stereology

All stereological analyses were carried out using a DM 6000B light microscope (Leica, Wetzlar, Germany) with motorized stage controlled by a computer-assisted stereology system (newCAST, Visiopharm, Horsholm, Denmark). Test fields for further analysis were gathered by systematic uniform random sampling from at least five different tissue blocks per animal. A point grid with an adjusted number of test points was projected onto each test field and the volume fraction of intra-alveolar edema fluid per unit volume of the parenchyma as the reference space (V_V_(ed/par)) was estimated by counting points hitting the intra-alveolar edema (P_ed_) and those points hitting the reference volume (P_ref_). The volume fraction was estimated by V_V_:= P_ed_/P_ref_ and converted to the total volume by multiplication with the reference volume [32],[33].

### Animal care

All animals received humane care in compliance with the European Convention on Animal Care and with the “Principles of Laboratory Animal Care” formulated by the National Society for Medical Research and the “Guide for the Care and Use of Laboratory Animals” prepared by the Institute of Laboratory Animal Resources, National Research Council, and published by the National Academy Press, revised 1996. The study was approved by the institutional ethics committee.

### Statistical analysis

All data are expressed as mean ± SD and were analyzed with the Statistical Program of Social Sciences (IBM SPSS Statistics, Version 20.0). Continous data were analyzed using ANOVA with repeated measures. For evaluation of data without repeated measures, standard analysis of variance was used. Statistical significance was assumed with a p-value <0.05.

## Results

**Figure 1:**
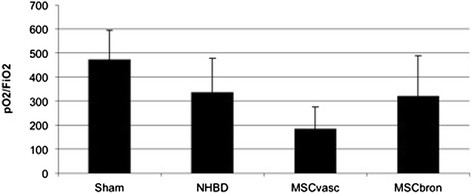
**Postischemic oxygenation (pO**_**2**_ **/F**_**i**_ **O**_**2**_ **) of transplanted pig NHBD lungs during the observation period of 4 hours.** Values are mean ± standard deviation. ANOVA (repeated measures): Sham versus MSCvasc: p = 0.001.

**Figure 2:**
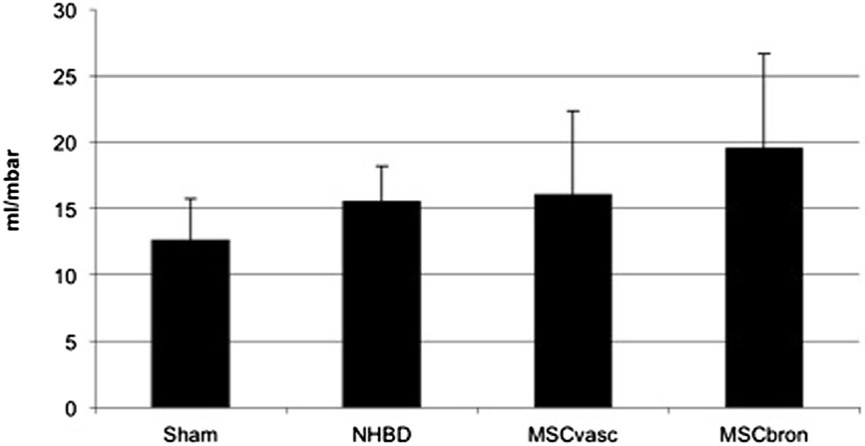
**Postischemic dynamic lung compliance of transplanted pig NHBD lungs during the observation period of 4 hours.** Values are mean ± standard deviation. ANOVA (repeated measures):. Sham versus MSCbron: p = 0.045.

**Figure 3:**
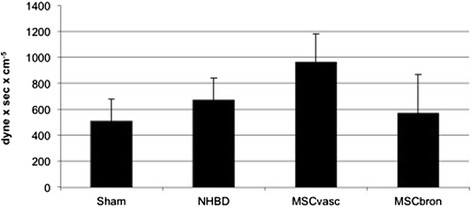
**Postischemic pulmonary vascular resistance of transplanted pig NHBD lungs during the observation period of 4 hours.** Values are mean ± standard deviation. ANOVA (repeated measures): Sham versus MSCvasc: p = 0.009.

**Figure 4:**
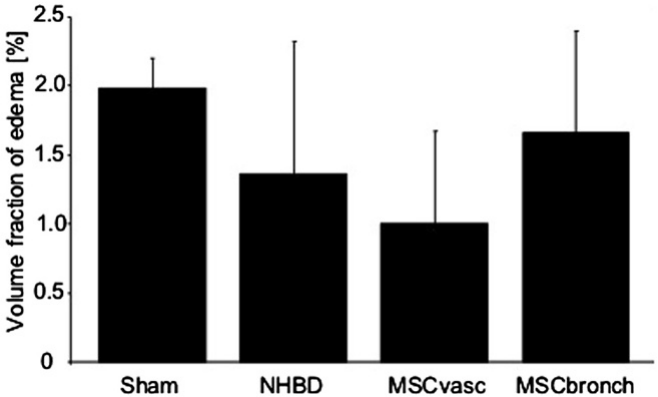
**Stereological quantification of intraalveolar edema (volume fraction referred to total lung parenchyma) of transplanted pig NHBD lungs after the observation period of 4 hours.** Values are mean ± standard deviation. Differences are statistically not significant (ANOVA : p = 0.251).

**Figure 5:**
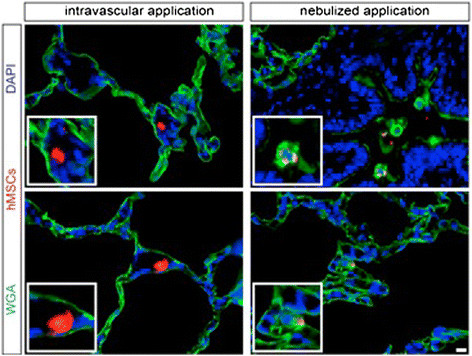
**Detection of transplanted hMSC in the corresponding compartments: following endovascular application in vessels of alveolar septa (left), following endobronchial (nebulized) application in the alveolar or bronchial lumen of native right NHBD lungs (right).** Scale bar = 10 μm.

**Figure 6:**
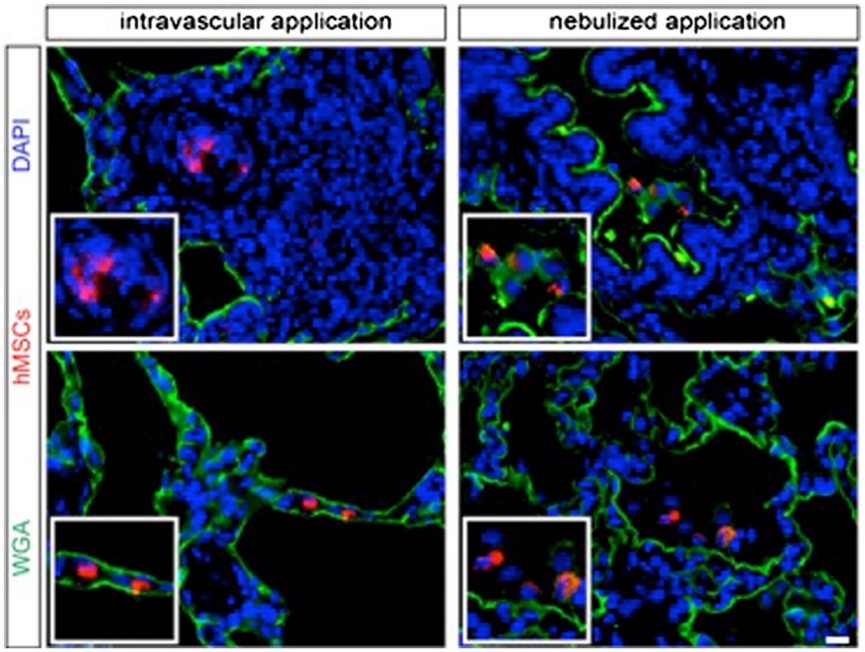
**Detection of transplanted hMSC in the corresponding compartments: following endovascular application in vessels of alveolar septa (left), following endobronchial (nebulized) application in the alveolar or bronchial lumen of transplanted and reperfused NHBD lungs (left).** Scale bar = 10 μm.

1. Functional analysis
  There was no mortality in the study groups. All Sham-operated animals presented with excellent oxygenation (pO_2_/F_i_O_2_: 472 ± 121 mmHg, Figure 1), dynamic lung compliance (DLC, 12.6 ± 3.1 ml/mbar, Figure 2) and pulmonary vascular resistance (507 ± 167 dynes*m^2^*sec^−5^, Figure 3). Compared with sham animals, non-heart-beating animals without MSC application presented with not significantly different outcome in terms of oxygenation (p = 0.087), PVR (p = 0,61) and DLC (p = 0.38) as we have shown previously (12). After vascular hMSC application (group 3), PVR (p = 0.09), oxygenation (p = 0.06) and compliance (p = 0.88) were comparable to NHBD without hMSC pretreatment (group 2), however PVR in group 3 (p = 0.009) was significantly higher and oxygenation significantly lower (p = 0.001) as compared to sham controls. In contrast, bronchial hMSC-application (group 4) resulted in PVR (p = 0.99) and oxygenation (p = 0.057) comparable with sham and showed significantly better values in dynamic lung compliance as compared to sham (p = 0.045).
2. Histological analysis
  The volume fraction of intraalveolar edema referred to total lung parenchyma [Vv(ed/par), Figure 4] was overall low with values of 0.0198 ± 0.002 (sham group 1), 0.0136 ± 0.0096 (group 2), 0.0100 ± 0.0068 (group 3) and 0.0166 ± 0.0073 (group 4, p = 0.123). Following endovascular application, corresponding hMSC in both preserved/unreperfused right lungs (Figure 5) and preserved/reperfused left grafts (Figure 6) were detected within vessels of the alveolar septa, whereas after endobronchial application, deposition of hMSC was proven to be in the alveolar lumen, respectively.

## Discussion

Ischemia-/reperfusion injury (IRI) of pulmonary allografts is characterized by increased alveolar epithelial and especially AE2-cell damage [34]–[37]. Furthermore, disturbances at the endothelial level are also considered to be a consequence of alveolar injury and, especially, of damage to AE2-cells [35]. This corresponds to the concept developed by Mason and Williams [38] who defined type II pneumocytes as the “defender of the alveolus”. The multiple functions of type II cells comprise surfactant synthesis, storage and secretion, ion- and water-transport, synthesis of growth factors etc. [39], therefore, AE2-cells are considered to be an essential element in stabilizing alveolar integrity and lung function. In accordance with several findings of our own group, strategies for improvements in pulmonary preservation were focused on maintenance of the blood-air barrier and especially type-II-cell functions [36],[37]. Aside from economically expensive application of exogenous surfactant in order to supplement the IRI-induced significantly altered intraalveolar surfactant pool [16],[40]–[42], an innovative cell therapy with recipient-based hMSC might represent a promising strategy due to paracrine, immunomodulating and tissue-remodeling properties of hMSC [18],[22]–[24] which include a trans-differentiation of hMSC into other cell types including epithelial cells and even AE2-cells [18],[19],[24]. In contrast to embryonic stem cells, bone marrow-derived hMSC have the distinct advantage of being abundant, easy to access with reasonable invasiveness and readily cultivated to a number sufficient for intrapulmonary application into the donor lung without ethical issues. With respect to the desirable potential effects on the epithelial layer, an application via the transbronchial route rather than a vascular approach is of theoretical advantage as the hMSC-homing-capacity and -duration depend on localisation and migration of delivered hMSC [25],[26]. In previously published findings of our group, an endobronchial application of iloprost into donor lungs resulted in significantly improved dynamic lung compliance and overall optimised lung preservation [12],[43],[44]. Correspondingly, in the current series of endobronchial application of hMSC, dynamic lung compliance was significantly higher as compared to Sham controls, and both pulmonary vascular resistance and oxygenation showed a clear tendency towards better values as compared to the transvascular route using the pulmonary artery which might indicate a beneficial short-term effect of this application mode even for hMSC application. However, hMSC will mainly exert the postulated beneficial effects in the intermediate and long-term phase following lung transplantation. Therefore, it was the aim of this pilot study to proof the general concept of intrapulmonary hMSC deposition into the NHBD lung by detection of administered stem cells in the recipient in a lung transplantation screening model with an initial reperfusion period of only 4 hours. In fluorescence microscopy we were able to document the explicit intrapulmonary deposition of the hMSC in both reperfused and unreperfused lungs although the total number of 50 million administered cells was rather low with regard to the total volume of a pig lung. In addition to the cited immediate effects in terms of significantly superior dynamic lung compliance, significant intermediate and long-term effects may therefore be postulated. As the immunogenicity of hMSC is low due to only low expression of MHC class I and II proteins in combination with lack of T-cell co-stimulating molecules CD80 und CD86, hMSC in general defy clearance by the recipient immune system and can be used in patients without HLA-matching [45],[46]. For the same reason we used xenogenic human MSC in this pig lung model as we are not able to isolate and expand porcine MSC so far. When the recipient-based hMSC would be obtained from waiting-list patients by regular bone-marrow aspiration and cultured to significant numbers in the increasingly extended waiting period for lung transplantation, then a significant immunologic response in the donor lung is clearly no issue [47].

Aside from the capability of hMSC to transform into other cell types, numerous paracrine factors like secretion of anti-inflammatory cytokines as IL-10 oder local growth factors as VEGF play a major role in regenerative medicine [18]. Depression in VEGF protein expression was shown by us to be an early event after IR and indicates increased alveolar epithelial and especially type II cell damage [35], therefore an increase in VEGF secretion and a reduced rate of apoptosis of lung parenchymal cells might be an important result of hMSC therapy [18],[23]. Also, an increased secretion of keratinocyte-growth factor (KGF) with corresponding proliferation of AE2 cells and subsequent increase in surfactant production [48],[49] in combination with direct paracrine effects on AE2 cells in terms of restoration of epithelial protein permeability by increased secretion of Angiopoietin-1 [50] are known beneficial effects of pulmonary hMSC therapy and might explain the experienced superior outcome in terms of dynamic lung compliance following endobronchial application of hMSC in our series. However, the optimal dose of hMSC, which is experimentally chosen in the area of 50 × 10^6^ per animal [27],[29] and which was also used in an ex-vivo perfused human lung experiment [48], has still to be finally determined.

The major cause of long-term morbidity and mortality in lung transplant recipients is the bronchiolitis obliterans syndrome (BOS) which is a form of chronic rejection characterized by an irreversible airway obstruction that affects about 50% of recipients surviving 5 and more years [1],[51]. In the pathogenesis of BOS, progressive fibroproliferation and accumulation of extracellular matrix culminate in a profound fibrotic obliteration of the airways [8],[52]. Recent findings suggest an important pathogenic role for donor-derived lung allograft-resident hMSC in fibroproliferative responses resulting in allograft remodeling [20],[21],[53]. As these allograft-resident hMSC were shown to have a significantly increased expression of several unique embryonic lung mesenchyme-associated transcription factors like FOXF1, HOXA5 and HOXB5 as compared with bone-marrow-derived hMSC, it is concluded that those cells are derived from donor embryonic mesenchyme and represent a locally resident tissue-specific progenitor cell [20]. As a hypothesis, application of recipient-specific bone marrow-derived MSC into the non-heart-beating donor lung might offer an innovative and easily applicable method to prophylactically initiate a specific autologous cell therapy in the pulmonary allograft before the onset of clinically significant ischemia-reperfusion injury (IRI). Whether the extent of chronic allograft dysfunction on terms of BOS can be positively influenced by this approach could easily be examined experimentally by bronchoalveolar lavage in the intermediate and long-term follow-up which is a standard method to obtain pulmonary hMSC by plastic adherence [21]. As a potential result, the onset and/or extent of the deleterious BOS might be influenced by specific donor-lung cell therapy and could result in improvement of long-term allograft function and patient survival which is currently significantly limited with increasing BOS status. The exact mode of action of transplanted hMSC in this promising approach still remains unclear. However, in most studies dealing with hMSC in the treatment of lung injury, engraftment rates of transplanted hMSC were <5% [50], suggesting that the magnitude of hMSC on repair appeared out of proportion to the number of donor-derived lung-specific hMSC. Therefore, the therapeutic benefit of transplanted hMSC might comprise a combination of paracrine effects that could stimulate the expansion, homing and differentiation of endogenous stem cells on one hand, and the direct differentiation of the transplanted autologous hMSC towards alveolar epithelial cells and other cell types on the other hand [47].

Recently, a novel form of chronic lung allograft dysfunction (CLAD) was described by Sato et al. [54],[55]. In contrast to BOS, which is characterized by small airway fibrosis and obstructive physiology, restrictive allograft syndrome (RAS) is characterized by peripheral lung fibrosis and restrictive physiology [54]. Importantly, RAS accounts for about 30% of CLAD, and survival of RAS-patients seems to be significantly shorter than that of BOS [54],[56]. The cause of RAS seems to be multifactorial and, similar to ARDS, an insult to the allograft such as infection or rejection leads to acute uncontrollable inflammation followed by fibrosis. In the pathogenesis of CLAD in general, activation of stromal resident cells such as epithelial cells and fibroblasts, is considered to be important [57], and specifically in RAS patients the stem cell population in terms of epithelial progenitors seems to be depleted over time since lung transplantation which might contribute to the increased vulnerability to damage or to the irreversible damage after an additional injury [58]. As the vicious cycle of immune-responsive cells and activated stromal resident cells through cytokines, chemokines and adhesion molecules may lead to irreversible tissue organization and fibrosis, an appropriate therapeutic target of CLAD in general and RAS in specific should not simply be the cause (i.e. rejection or infection), but instead the final common pathway of uncontrollable inflammation and subsequent fibrosis which seems to be related to the depletion of the stem-cell population in the allograft [55],[58].

## Conclusion

A specific donor-lung cell therapy with effective pre-ischemic intrapulmonary hMSC transplantation might be a promising therapy to positively influence the onset and/or the extent of both the deleterious bronchiolitis obliterans (BOS) and restrictive allograft syndrome (RAS). Most importantly, such a hMSC-based therapy has a significant safety advantage as no adverse differentiation of transplanted MSC has been observed, as opposed to potential teratoma formation from pluripotent cells and their derivatives [59],[60].

Consequently, according to a well recognized review by Moodley et al. [61], studies like our current work who have the potential to elucidate further pathways that mediate the action of bone-marrow derived mesenchymal stem cells are generally considered to be “vital to future therapeutic strategies in the treatment of lung disease”. However, limitations of this study comprise the short reperfusion period of 4 hours in this acute phase screening model, the low number of transplanted hMSC and the missing vitality testing of transplanted MSC. All aspects will be specifically addressed in future experiments in a chronic lung transplantation modell which will be established in our group.

## Authors’ original submitted files for images

Below are the links to the authors’ original submitted files for images. Authors’ original file for figure 1 Authors’ original file for figure 2 Authors’ original file for figure 3 Authors’ original file for figure 4 Authors’ original file for figure 5 Authors’ original file for figure 6

## Abbreviations

AE2: Alveolar epithelial type 2
BM: Bone marrow
BOS: Bronchiolitis obliterans syndrome
CLAD: Chronic allograft dysfunction
DAPI: 4’,6-diamidino-2-phenylindole
DLC: Dynamic lung compliance
hMSC: Human mesenchymal stem cell
KGF: Keratinocyte growth factor
LPD: Low-potassium dextrane
LTx: Lung transplantation
NHBD: Non heart beating donor
O.C.T: Tissue-Tek® O.C.T™, Sakura Inc.. “Formulation of watersoluble glycols and resins, providing a convenient specimen matrix for cryostat sectioning”
PBS: Phosphate buffered saline
PEEP: Positive end-exspiratory pressure
PVR: Pulmonary vascular resistance
RAS: Restrictive allograft syndrome
VEGF: Vascular endothelial growth factor
WGA: Wheat-germ-agglutinin
