# Erratum: Mesenchymal stem cell pretreatment of non-heart-beating-donors in experimental lung transplantation

**DOI:** 10.1186/s13019-014-0198-1

**Published:** 2015-05-21

**Authors:** Thorsten Wittwer, Parwis Rahmanian, Yeong-Hoon Choi, Mohamed Zeriouh, Samira Karavidic, Klaus Neef, Astrid Christmann, Tanja Piatkowski, Anke Schnapper, Matthias Ochs, Christian Mühlfeld, Anja Sterner-Kock, Maria Guschlbauer, Florian Hofmaier, Alexandra C Maul, Thorsten Wahlers

**Affiliations:** Department of Cardiothoracic Surgery, Heart Center, University of Cologne, Kerpener Strasse 61, 50924 Cologne, Germany; Center of Molecular Medicine, University of Cologne, Cologne, Germany; Institute of Functional and Applied Anatomy, Hannover Medical School, Biomedical Research in Endstage and Obstructive Lung Disease Hannover (BREATH), Member of the German Center for Lung Research (DZL), Hannover, Germany; Cluster of Excellence Rebirth (From Regenerative Biology to Reconstructive Therapy), Hannover, Germany; Center for Experimental Medicine, University of Cologne, Cologne, Germany

Following publication of this manuscript [1] it was discovered that several authors were inadvertently omitted from the author list. Anja Sterner-Kock, Maria Guschbauer, Florian Hofmaier and Alexandra Maul have now been added to the correct author list above. The authors apologise for any inconvenience this may cause.
