# Supplementary figures and images for: Mesenchymal stem cell pretreatment of non-heart-beating-donors in experimental lung transplantation
Source: J Cardiothorac Surg. 2014 Sep 2;9:151. doi: 10.1186/s13019-014-0151-3 (PMC4169637; doi:10.1186/s13019-014-0151-3)

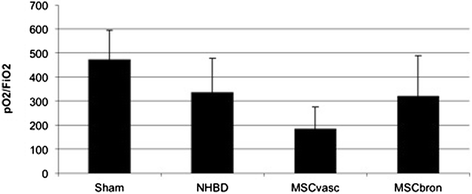

Supplement: Supplementary file 1 — Authors’ original file for figure 1 [file 13019_2014_151_MOESM1_ESM.gif]

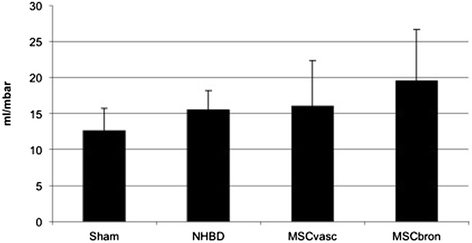

Supplement: Supplementary file 2 — Authors’ original file for figure 2 [file 13019_2014_151_MOESM2_ESM.gif]

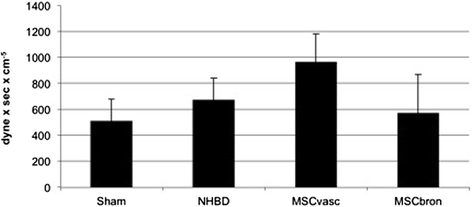

Supplement: Supplementary file 3 — Authors’ original file for figure 3 [file 13019_2014_151_MOESM3_ESM.gif]

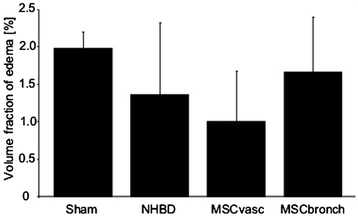

Supplement: Supplementary file 4 — Authors’ original file for figure 4 [file 13019_2014_151_MOESM4_ESM.gif]

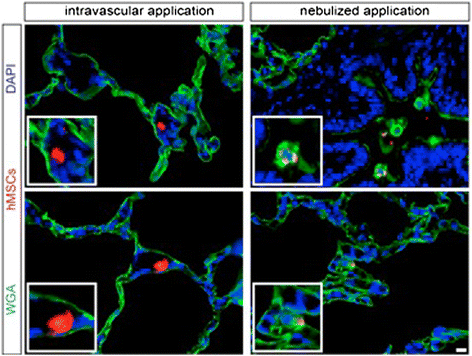

Supplement: Supplementary file 5 — Authors’ original file for figure 5 [file 13019_2014_151_MOESM5_ESM.gif]

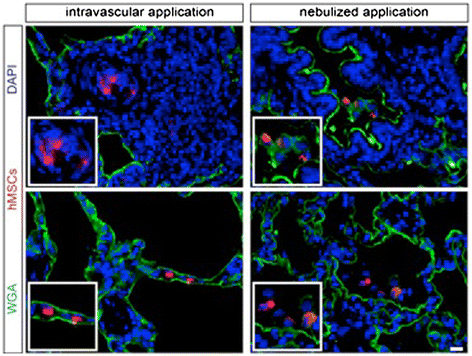

Supplement: Supplementary file 6 — Authors’ original file for figure 6 [file 13019_2014_151_MOESM6_ESM.gif]
